# The Acute Impact of Smoking One Cigarette on Cardiac Hemodynamic Parameters

**DOI:** 10.4021/cr24e

**Published:** 2011-03-25

**Authors:** Khalid Abou Farha, Ramy AbouFarha, Marc Bolt

**Affiliations:** a PRA-International, Institute for Clinical Pharmacology, Groningen, Netherlands; b Dr. Nassau College, Grammar School, Assen, Netherlands

**Keywords:** Smoking, Cigarette, Cardiac hemodynamic parameters

## Abstract

**Background:**

The acute impact of tobacco smoking on the cardiac hemodynamic parameters and its pathological implication in the process of arterial atherosclerosis need further exploration. This investigation was purposed to assess the acute impact of tobacco smoke on blood pressure and cardiac hemodynamic parameters.

**Methods:**

Using an Ultrasonic Cardiac Output Monitor, and DINAMAP Pro 400 Series V2 blood pressure monitor, several cardiac hemodynamic parameters and the blood pressure were assessed in 14 smokers, 11 females and 3 males, at 2 time points, before and after smoking of one cigarette. Data, in terms of ratio of the means and 95% confidence interval were analyzed using ANOVA.

**Results:**

Single-subject design in which the subject has served as his/her own control has been used. Tobacco smoking led to statistically significant acute increase in the means of all hemodynamic parameters, except for heart rate in female subjects, as compared to the means obtained before smoking.

**Conclusions:**

Cigarette smoking induces acute non-physiologic alteration in cardiac outflow forces, exposing the aortic valve and arch to mechanical injury that might be implicated in initiating and promoting the process of aortic arch atherosclerosis and associated pathological lesions.

## Introduction

The use of tobacco leaf for treatment of various illnesses and sometimes for its pleasurable effect was introduced by the Native Americans to Columbus as early as the 15th of October 1492. However the use of tobacco as cigarette is a 20th century phenomenon [1].

Tobacco smoking is the leading preventable cause of death (40%) [2]. Cardiovascular diseases (CVD) have been shown to be the leading cause of death from smoking. In the year 2003 there were 4.83 million premature deaths in the world attributable to smoking. CVDs caused 1.69 million of these [3]. It is estimated that 26% of people aged 15 years and older in the EU, or about 100 million people, are current daily smokers and about 50 million of these will die prematurely from smoking unless they quit [4]. Moreover, it has been estimated that by 2030 tobacco is going to kill 8 million people a year [5].

The average cigarette contains 6 - 11 mg nicotine and delivers about 1 - 3 mg nicotine systemically to the smoker [6]. The inhaled cigarette smoke is rapidly absorbed from small airways and alveoli reaching a blood maximum concentration after 5 - 8 minutes and declines over the next 20 minutes due to tissue distribution [7, 8]. The intake of nicotine during smoking depends on the puff volume, the extent of dilution with room air, rate of puffing and extent of swallowing nicotine laden saliva [7]. Nicotine is eliminated mainly by hepatic metabolism (80% - 90%) and also by the kidney with a half-life elimination time of 2 hour [6, 7].

The effect of smoking on cardiovascular system is multifactorial. Cigarette smoking induces and promotes a progressive interlacing complex process that might end up with vascular occlusion, myocardial infarction and stroke. Tobacco smoking has been identified as a strong and independent cause of vascular atherosclerosis and is reported as a major cause of occlusive arterial diseases (including coronary arteries), aortic valve stenosis and thoracic aortic dilatation and aneurysm, specifically in the aortic arch [9-11]. Cigarette smoking-induced atherosclerosis is partly attributed to the vascular endothelial damage caused by the free radicals and reactive oxygen species (ROS) in the cigarette smoking and by decreasing the nitric oxide (NO) bioactivity in vascular endothelium as well as platelet derived NO release [2]. NO is a regulator of the mitochondrial ROS detoxification system. Reduced levels of NO lead to ROS high levels resulting in lipid per oxidation and damage to cell membranes, protein and DNA and ultimately result in vascular endothelial cell dysfunction and death [12]. Although the entire vascular endothelium is exposed to the above-mentioned tobacco-induced humoral responses, atherscelerotic lesions develop preferentially at certain sites including the inner curvatures of the arteries, like aortic arch. This suggest the presence of other local factors that promote disease susceptibility like disturbed cardiac hemodynamic forces that might lead to non-physiologic mechanical stress effects [10, 13].

To the best of our knowledge, the acute change in the cardiac hemodynamic profile induced by tobacco smoking and its impact on the cardiovascular integrity are scarcely addressed in the literature. Given that, we decided to conduct this pilot cross-sectional investigation to explore the acute effect of smoking one cigarette on a number of cardiac hemodynamic parameters and blood pressure.

## Methods

### Subjects

We performed a pilot cross-sectional study to investigate the acute effect of cigarette smoking on a number of cardiac hemodynamic parameters and blood pressure. Fourteen young healthy subjects, 11 females and 3 males, aged 24 - 51 years served as study population. All subjects were regular smokers (10 - 20 cigarettes per day for 5 - 25 years). All subjects included in this pilot study are employees of the clinical pharmacology institute of PRA-international, Groningen, the Netherlands. A written consent form to publish the study data has been obtained from all participated subjects. All female participants were using hormonal contraception. No other medications including over the counter medications were used by any of the participated subjects. None of the subjects had a history of any relevant medical disease. The cardiac hemodynamic parameters and brachial blood pressure were assessed at 2 time points, after 3 - 5 minutes rest, before smoking (at least 2 hours refrain from smoking, served as a baseline value) and up to 15 minutes after smoking.

### Echocardiographic assessments

In this investigation we used the Ultrasonic Cardiac Output Monitor (USCOM Pty Ltd, Coffs Harbour, NSW, Australia). USCOM is a well validated non-invasive device that determines cardiac output by a continuous-wave (C-W) Doppler Ultrasound [13]. The USCOM machine displays many real-time data about cardiac hemodynamic through evaluating the aortic or pulmonary blood flow as it leaves the heart. Validated internal algorithms calculate the diameter of the aortic and pulmonary valve based upon the patient’s height and weight. Using a 2.2 MHz USCOM transducer we assessed the following systolic flow profiles: heart rate (HR); cardiac output (COP); cardiac index (CI); stroke volume (SV); stroke volume index (SVI); aortic peak velocity (VpK); minute distance (MD); ejection time percentage (ET%); and mean pressure gradient (Pmn). In this regard, the suprasternal notch acoustic window has been selected to scan, for at least one minute, the flow profile of several cardiac cycles (one cardiac cycle is ± 0.8 seconds) obtained from each subject in the supine position. The average of values obtained for each parameter was subsequently considered in the evaluation. To avoid the possibility of inter-observer variation and tracing different blood flow strengths and subsequently signaling different hemodynamic profiles, one investigator made the assessment by placing the transducer on the same point of optimal acoustic signal displayed as pointy peak on the USCOM screen. As for assessment of blood pressure, the well validated and widely used DINAMAP Pro 400 Series V2, GE medical system was used. Brachial blood pressure, Systolic (SBP) and Diastolic (DBP) were measured. Derived variables were calculated as follows: Mean arterial blood pressure (MAP) = 1/3 SBP + 2/3 DBP and Pulse Pressure (PP) = SBP - DBP.

### Statistical analysis

Single-subject design in which the subject served as his/her own control has been used. The data were analyzed with ANOVA. To analyze the hemodynamic parameters, a mixed model was applied using treatment (pre- or post-smoking) and gender by treatment as fixed effects and subject as a random effect. The individual values were (natural) LOG transformed before statistical analysis and the results were back transformed. From this analysis estimates of least square means and the ratio of post- and pre-smoking (with 95% confidence interval) were obtained. Estimates were made for the entire group and for male and female groups to detect gender related differences. The probabilities to obtain the observed data, or still more extreme data when null hypothesis (H0) was true are presented as P values.

## Results

**Figure 1:**
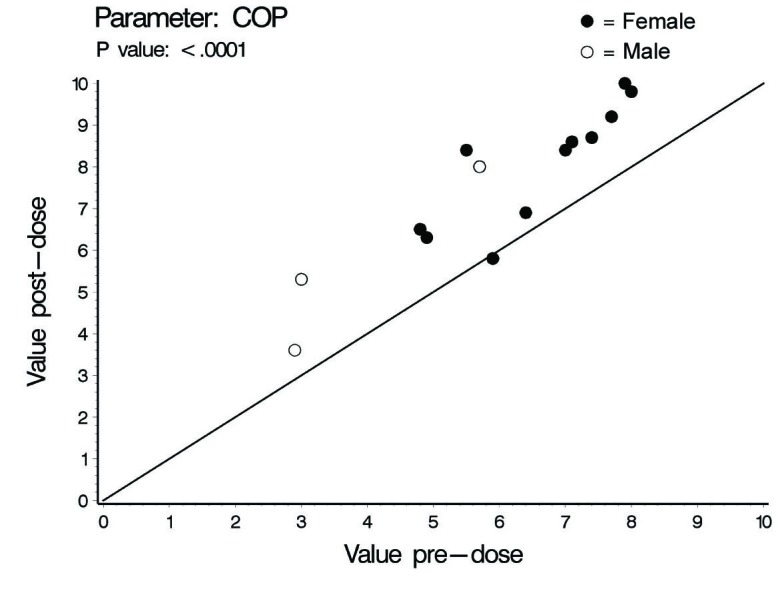
Cardiac output (COP), individual values.

In this investigation a statistically significant difference at the level of ≤ 0.02 between all cardiac hemodynamic values (except for HR in female subjects) obtained before and after smoking has been observed. In spite of the small sample size (14 subjects) used in this pilot cross-sectional study, there was an obvious increase in the means of the assessed hemodynamic parameters obtained after smoking. At the individual subject level, 4 female subjects demonstrated no changes in some hemodynamic parameters obtained after smoking as compared to base line values; Vpk, Pmn, MD, ET% and COP in one subject; Vpk, Pmn and SVI in one subject; Vpk and HR in one subject and HR in one subject. As for the blood pressure values and derived variables, we found respectively in male and female subjects increase of 8 and 3 mmHg in SBP; increase of 1.6 and 2.7 mmHg in DBP; increase of 4 and 2.8 mmHg in MAP; and increase of 828 and 138 in the HR-PP product (pulsatile stress index) during one minute. Tables 1, 2 and 3 and Figures 1 through to 6 depict the obtained results.

**Table 1:** Statistical Analysis Parameters Measured Before and After Smoking (All N = 14)

Parameter	LS means		Ratio (after/before)			
			Geometric mean	Confidence interval 95%		Pr > |t| *
	Before	After		Lower	Upper	
VPK	1.04	1.23	1.18	1.08	1.30	0.0019
PMN	2.05	2.83	1.38	1.20	1.59	0.0003
HR	69.7	76.0	1.09	1.04	1.14	0.0013
MD	14.5	19.6	1.35	1.23	1.48	< 0.0001
ET%	34.9	40.5	1.16	1.12	1.20	< 0.0001
SV	69.9	86.0	1.23	1.15	1.32	< 0.0001
SVI	35.3	43.2	1.22	1.14	1.32	< 0.0001
COP	4.89	6.51	1.33	1.22	1.46	< 0.0001
CI	2.45	3.30	1.35	1.22	1.49	< 0.0001
SBP	130	135	1.04	0.98	1.10	0.1987*
DBP	81.5	83.6	1.03	0.98	1.07	0.1968*
PP	48.1	51.1	1.06	0.96	1.18	0.2327*
MBP	97.8	101	1.03	0.98	1.08	0.1815*
HR x PP	3351	3882	1.16	1.01	1.32	0.0323

The parameters were analyzed with ANOVA, treatment (pre- or post-smoking) and treatment* gender as fixed factors and subject as a random factor.

The values were (natural) LOG transformed before (and back transformed after) statistical analysis.

*indicates if the P-value is above 0.05 (no alpha level was pre-defined).

**Table 2:** Statistical Analysis Parameters Measured Before and After Smoking (Female N = 11)

Parameter	LS means		Ratio (after/before)			
			Geometric mean	Confidence interval 95%		Pr > |t| *
	Before	After		Lower	Upper	
VPK	1.21	1.39	1.16	1.06	1.26	0.0032
PMN	2.61	3.39	1.30	1.14	1.48	0.0010
HR	75.8	78.4	1.03	0.99	1.08	0.1078*
MD	19.9	24.6	1.24	1.14	1.35	0.0002
ET%	43.0	46.8	1.09	1.05	1.12	0.0001
SV	85.7	102	1.19	1.12	1.27	< 0.0001
SVI	44.9	53.5	1.19	1.11	1.27	< 0.0001
COP	6.50	7.93	1.22	1.12	1.33	0.0002
CI	3.40	4.18	1.23	1.12	1.35	0.0004
SBP	129	132	1.02	0.97	1.08	0.4344*
DBP	75.6	78.3	1.03	1.00	1.08	0.0760*
PP	53.1	53.1	1.00	0.91	1.10	0.9937*
MBP	93.5	96.2	1.03	0.98	1.07	0.1924*
HR x PP	4029	4167	1.03	0.92	1.17	0.5579*

The parameters were analyzed with ANOVA, treatment (pre- or post-smoking) and treatment* gender as fixed factors and subject as a random factor.

The values were (natural) LOG transformed before (and back transformed after) statistical analysis.

*indicates if the P-value is above 0.05 (no alpha level was pre-defined).

**Table 3:** Analysis Parameters Measured Before and After Smoking (Male N = 3)

Parameter	LS means		Ratio (after/before)			
			Geometric mean	Confidence interval 95%		Pr > |t| *
	Before	After		Lower	Upper	
VPK	0.901	1.09	1.21	1.03	1.43	0.0256
PMN	1.61	2.37	1.47	1.14	1.88	0.0061
HR	64.0	73.6	1.15	1.06	1.25	0.0025
MD	10.5	15.5	1.48	1.25	1.74	0.0002
ET%	28.4	35.0	1.24	1.16	1.32	< 0.0001
SV	57.1	72.7	1.27	1.13	1.44	0.0010
SVI	27.7	34.9	1.26	1.11	1.43	0.0021
COP	3.67	5.34	1.45	1.24	1.71	0.0003
CI	1.76	2.62	1.49	1.24	1.77	0.0004
SBP	131	139	1.06	0.95	1.17	0.2877
DBP	87.7	89.3	1.02	0.95	1.10	0.6074
PP	43.6	49.1	1.13	0.94	1.36	0.1829
MBP	102	106	1.03	0.95	1.13	0.3968
HR x PP	2788	3616	1.30	1.03	1.64	0.0325

The parameters were analyzed with ANOVA, treatment (pre- or post-smoking) and treatment* gender as fixed factors and subject as a random factor.

The values were (natural) LOG transformed before (and back transformed after) statistical analysis.

*indicates if the P-value is above 0.05 (no alpha level was pre-defined).

**Figure 2:**
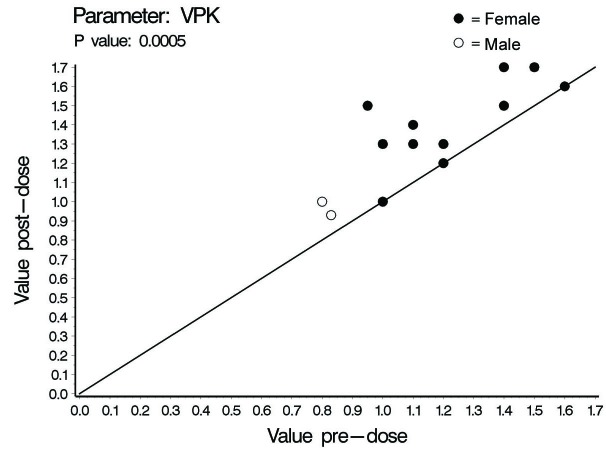
Aortic peak velocity (VpK), individual values.

**Figure 3:**
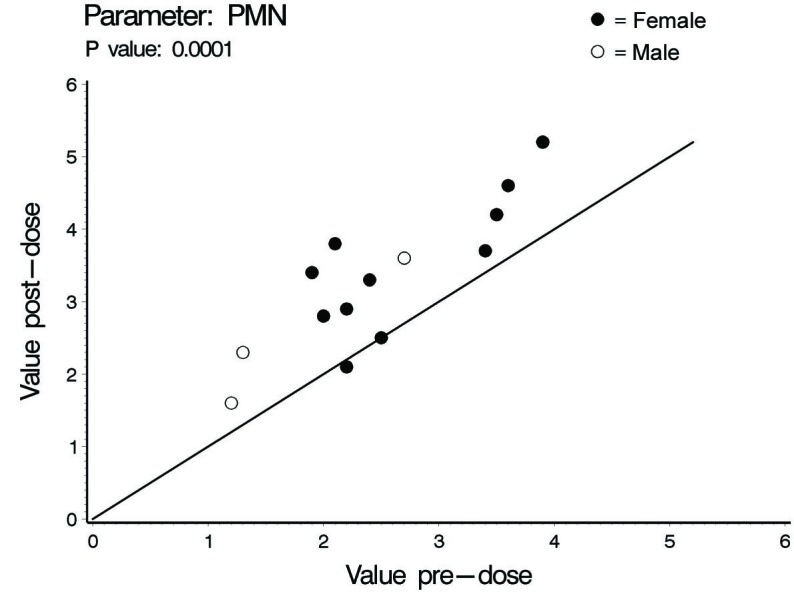
Aortic valve mean pressure gradient (Pmn), individual values.

**Figure 4:**
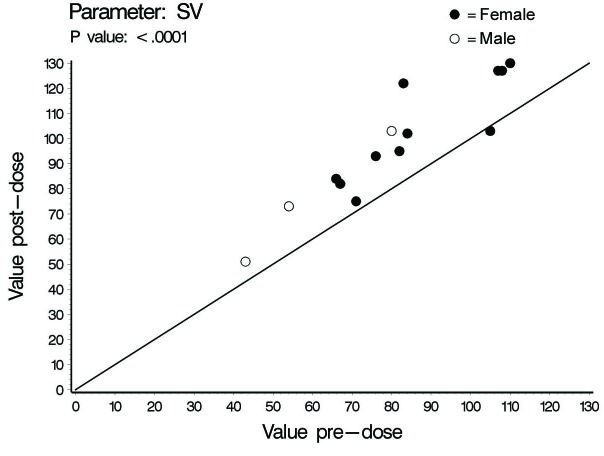
Stroke volume (SV), individual values.

**Figure 5:**
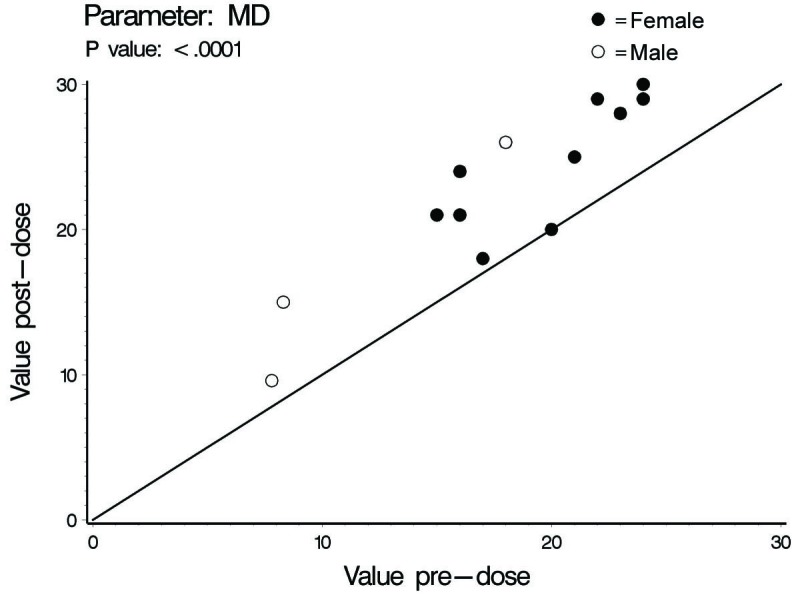
Minute distance (MD), individual values.

**Figure 6:**
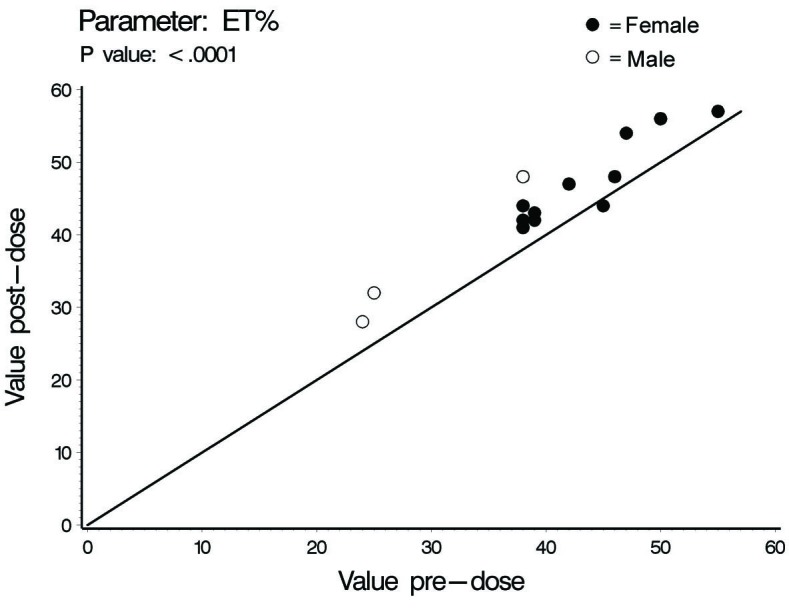
Ejection time percentage (ET%), individual values.

## Discussion

The concentration of nicotine in arterial blood after smoking one cigarette ranges between 20 - 100 ng/ml. In smokers, the mean nicotine boost after smoking a cigarette is 10.9 ng/ml blood. Given a 2 h half-life time for nicotine, accumulation is predicted over 8 h (4 half-life times) of regular smoking with persistence of high blood nicotine level for 8 h after cessation of smoking. This indicates that regular smokers would have increment in blood nicotine concentration as the day progress with significantly high nicotine plasma levels during sleep [7]. The cardiovascular endothelial lining of regular smokers is, therefore, continuously exposed to ascending high nicotine concentration. This pilot study showed some interesting findings. In spite of the low sample size which is expected to decrease the power of statistical analysis, smoking one cigarette was associated with significant increase in the COP, Vpk and Pmn as compared to values obtained at pre-smoking time point. The increases observed in males and females were respectively (45% and 22%), (21 % and 16 %) and (47 % and 30%) for COP, Pmn and Vpk. The increase in these variables even across anatomically normal aortic valve can cause highly disturbed and turbulent aortic blood flow [15, 16]. Turbulent blood flow contributes to a variety of arterial pathological effects. Unlike the athero-protective normal laminar shear stress, turbulent or oscillatory blood flow induces and promotes the development of atherosclerotic plaque formation especially in atherosclerosis susceptible areas exposed to a weak net hemodynamic athero-protective shear stress, e.g., aortic arch [17]. Exposure of vascular endothelial cells (VECs) to turbulent oscillatory shear stress is associated with prolonged reactive oxygen species generation (ROS) and H_2_O_2_ production. In addition, turbulent shear stress fails to stimulate expression of endothelial nitric oxide synthase and Mn superoxide dismutase which form an important antioxidant defense system [17-19]. Protracted turbulent shear stress disturbs the balance between ROS production and ROS neutralizing enzymes which becomes tipped towards oxidative status where the produced nitric oxide is insufficient to counterbalance the increased ROS [17]. This results in increased expression of vascular cell adhesion protein molecules that bind to inflammatory cells and endothelial cell loss, apoptosis and desquamation creating leaky junctions over the endothelium that increase endothelium permeability to macromolecules including LDL [18, 20, 21]. Turbulent flow up-regulates the production of endothelin-1 that acts as a vasoconstrictor and vascular smooth muscle cell (VSMC) mitogen, a key factor in the development of vascular wall hyperplasia [19]. It also up-regulates endothelium derived bone morphogenic proteins (BMPs) suggested to induce vascular calcification [22]. The ultimate end results will be development of atherosclerosis. The observed post one cigarette smoking increase in SV, 27% and 19% in males and females respectively, reflects increase in the intraventricular pressure. Giving the fact that myocardium is only perfused during diastole, the resultant increase in intraventricular pressure together with the observed increase in the systolic ejection time (ET%), 24% in males and 9% in females, might limit the adequate coronary perfusion and myocardium oxygen supply. In addition, the observed increase in MD, 48% in males and 24% in females, Vpk and SV indicate increased myocardial contractility and thence increased myocardial oxygen demands. This together with the well-established effect of smoking in inducing arterial oxygen de-saturation [23, 24] aggravate the negative impact of smoking-induced limitation of myocardial perfusion.

The difference between all assessed blood pressure variables, except for the product of HR and PP in male subjects, did not reach statistical significance. This might be attributed to low power of the test reasoned by the small sample size. Nevertheless, the observed difference might bear some pathophysiological and clinical importance, particularly in regular smokers where the CVS is continuously exposed to cumulative plasma nicotine levels. Under normal conditions arterial vascular walls are continuously exposed to physiologic levels of cyclic strains and pulsatile distension imposed by heart propulsion (stroke volume) and systolic- diastolic blood pressure phases. However, variation within normal physiologic pulsatile distension of arterial wall does not exceed 10% - 12% [25]. Non-physiologic chronic cyclic stress stimulates the expression and activity of a number of proteolytic enzymes including matrix metalloproteinases (MMP) and extracellular matrix degrading enzymes, that cleave extracellular matrix as well as non-matrix substances resulting in increased permeability to macromolecules including LDL. Cyclic strain also increases ROS generation and cleaved Caspase expression, a pro-apoptotic event. These changes result in endothelial cell dysfunction, detachment and apoptosis [20, 26]. On the other hand, mechanical stretch of the vessel wall was found to up-regulate angiotensin II receptors (AT1 receptors) and consequently sensitize VSMC to angiotensin II, a potent vasoconstrictor and extracellular matrix synthesis inducer [27]. In addition, non-physiologic increased cyclic stretch activates VSM and promotes VSMC DNA synthesis and proliferation leading to increased wall thickness and decreased vascular wall compliance [25, 28]. The pathophysiological mechanisms leading to initiation and development of arterial atherosclerosis are similar to those involved pathogenesis of aortic stenosis [11, 29]. In this regard, increased mechanical stress and hemodynamic forces resulting from increased intraventricular pressure might prematurely initiate aortic valve injury and promote valve infiltration with inflammatory cells. The reported up-regulation of MMP, BMPs and AT-1 receptors in the thickened stenotic hypo mobile aortic valve [29] might underline the role of smoking-induced nonphysiologic mechanical stress in initiating the process of aortic valve stenosis. From a clinical point of view, increased amplitude and frequency of pulsatile stress, indicated by the product of HR and PP, as observed in the male subjects has been reported as an independent determinant of coronary heart disease (CHD) [30]. In this pilot study we observed increase of 8 and 3 mmHg in SBP and increase of 1.6 and 2.7 mmHg in DBP in male and female respectively. A 2 to 3 mmHg difference in brachial BP was also reported to make 20% to 30% difference in cardiovascular risk [31]. In a meta analysis of individual data obtained from one million adults, long term increase of 10 mmHg in SBP or 5 mmHg in DBP was found to be associated with 40% higher risk of stroke death and 30% higher risk of death from coronary heart disease. Even increase of 2 mmHg in SBP can be associated with 10% higher stroke mortality and 7% mortality from ischemic heart disease [32].

In this study there was some differences in the post smoking hemodynamic variables obtained from males and females. The effect of smoking one cigarette on the studied hemodynamic parameters was more pronounced in male subjects. This might be explained by previous reports. Koylu and associates [33] observed that chronic nicotine administration to rats was associated with higher nicotine acetylcholine receptor densities in male but not female rat brains. In this context, studying the impact of smoking on the neuronal intra-cardiac nicotine acetylcholine receptors might add some insights into the acute cardiac pathophysiology of smoking.

Finally, it is worth mentioning that the results of this pilot study represent the acute impact of nicotine boost delivered by smoking one cigarette. Assessments in this exploratory investigation was performed at random time points without considering the number of previously smoked cigarettes, effect of certain confounders that might alter the blood nicotine concentration like relation to meal, puff volume and depth of inhalation, rate of puffing and type and strength of used contraceptives in females. Considering such confounders a large sample-sized study, to increase the power of analysis, would give more insight into the acute nonphysiologic hemodynamic changes of smoking that seem to initiate the process of atherosclerotic process through mechanical injury of atheroprone vascular endothelium, like aortic arch.
